# An Optimization Algorithm for Multipath Parallel Allocation for Service Resource in the Simulation Task Workflow

**DOI:** 10.1155/2014/507517

**Published:** 2014-05-12

**Authors:** Zhiteng Wang, Hongjun Zhang, Rui Zhang, Yong Li, Xuliang Zhang

**Affiliations:** ^1^PLA University of Science & Technology, Nanjing 210007, China; ^2^Nanjing Artillery Academy, Nanjing 210110, China

## Abstract

Service oriented modeling and simulation are hot issues in the field of modeling and simulation, and there is need to call service resources when simulation task workflow is running. How to optimize the service resource allocation to ensure that the task is complete effectively is an important issue in this area. In military modeling and simulation field, it is important to improve the probability of success and timeliness in simulation task workflow. Therefore, this paper proposes an optimization algorithm for multipath service resource parallel allocation, in which multipath service resource parallel allocation model is built and multiple chains coding scheme quantum optimization algorithm is used for optimization and solution. The multiple chains coding scheme quantum optimization algorithm is to extend parallel search space to improve search efficiency. Through the simulation experiment, this paper investigates the effect for the probability of success in simulation task workflow from different optimization algorithm, service allocation strategy, and path number, and the simulation result shows that the optimization algorithm for multipath service resource parallel allocation is an effective method to improve the probability of success and timeliness in simulation task workflow.

## 1. Introduction

With the development of web technology, web service technology, SOA, grid, and cloud computation, there is a great revolution in modelling and simulation field. A framework of modelling and simulation based on simulation service, that is, service oriented modelling and simulation, is being formed. Service oriented simulation task framework from service oriented idea depends on information grid infrastructure with unified simulation description, access, and share standard to complete simulation task with the way of dynamic creation and running in the simulation resource interlink and share environment. Compared with traditional simulation framework, one of the service oriented modeling and simulation framework main characteristics is supporting dynamic creation and running simulation application and dynamic integration and running simulation in network by calling simulation service resource according to the simulation task workflow need.

In service oriented military modeling and simulation field, it is important to improve the probability of success and timeliness in simulation task workflow. Many researchers have done lots of research on how to improve the probability of success and timeliness in service composition in many stages. Some researchers try to improve the probability of simulation task workflow work success instead of failed service [1–3], but this method does not consider the timelessness of workflow. Therefore, it will produce extra time charge when the called service cannot respond or makes a mistake. Artificial intelligence (AI) planner is also applied to solve service composition combinatorial optimization problem [4–7]. But for the reason that it could immensely increase coupling between AI planner and other procedures when the special AI planner is used to solve automatic service composition problem, it will lead to limiting its web composition application filed. In addition, there are many differences in AI planner for applicability and designs performance, and this reason will result in biased service composition scheme and larger composition charge. Yajuan Song proposes a blend web service composition scheme based on buffer pool [8], in which dynamic service composition scheme is saved in buffer pool and user can use the composition scheme as well as use predefined workflow. But the composition algorithm should be improved. ZHANG Bo investigates a service composition algorithm based on sub-Web service, in which the multi-input and multioutput parameter of web service is divided into sub-Web service with multi-input and single output parameters. In this way, it reduces dependencies of a Web service for multiple outputs with higher composition efficiency. In this way, we reduce dependencies of a Web service on multiple-output with higher composition efficiency; however, there exists redundancy in the composition route. Genetic algorithm (GA) and particle swarm optimization (PSO) [9] are typical evolution algorithm in service composition optimization filed, and PSO is more effective than GA with advantages such as too few arguments specified and the faster convergence speed. However, the local particle in the swarm is easy to be regarded as global best position leading to quickly converging to local best value. Zheng et al. [10] and Zeng et al. [11] use objective programming method to find the best path satisfied with condition by building the objective function with the condition and the probability of success and time requisition. The shortcomings of which may not complete the workflow requests.

Xiaohao et al. propose parallel allocation algorithm for service [12], in which one node starts many services to improve the probability of success and time requests in the simulation task workflow. The method gets good result but there exists exception probability in large-scale simulation task. Therefore, based on service parallel allocation in [12], this paper extends the scheme and proposes an optimization algorithm for multipath service resource parallel allocation scheme to further improve the probability of simulation task workflow work success and time and solve service scheduling problem.

The following section will investigate the systematic structure of simulation task body from two perspectives, that is, the reference model and the framework structure of simulation task body.

In service oriented military model and simulation field, it is important to ensure the timeliness and the probability of success in the simulation task workflow or it will greatly affect the workflow work efficiency and even lead to workflow which cannot complete the task. In fact, it could improve the timeliness and the probability of success by the optimization of the service composition in simulation task workflow. However, many optimization algorithms have deficiencies in solving this problem. For this reason, this paper proposes an optimization algorithm for multipath service resource parallel allocation for service resource to improve the timeliness and the probability of success in the simulation task workflow.

## 2. The Description of Service Composition Problem

### 2.1. The Concept of Service Scheduling and Simulation Task Workflow Framework

**Figure 1:**
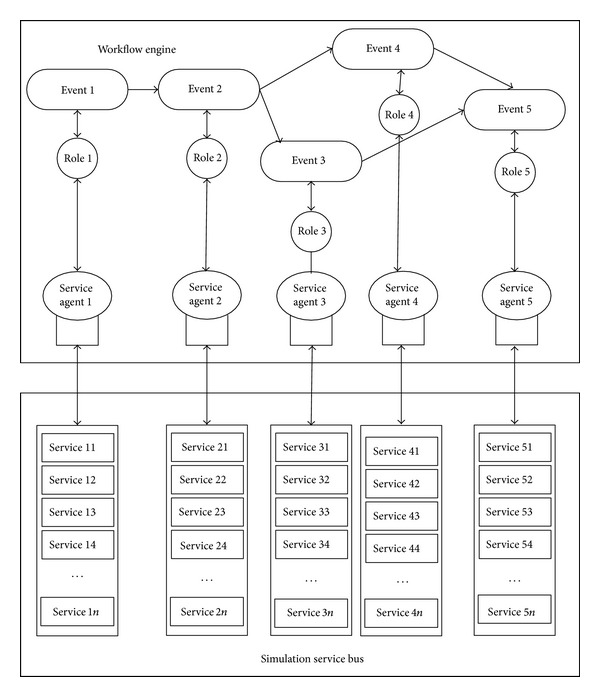
Simulation task workflow running framework.

The concept of workflow originated from the organization of production and office automation field, in which work was separated into well-defined task, role, and the work is executed and monitored by specific rule and process to improve work probability and reduce production costs and raise enterprise competitive power. In military service oriented model and simulation field, workflow idea is introduced to arrange the role produce specific event under the simulation plot rule in simulation task. The event of role is related to simulation service agent, which is a service set and could call the simulation service by simulation service bus. The specific simulation service in service sets is eventual execution unit, and the purpose of service scheduling is to find the best path to allocate the simulation service in service sets to specific event of role in simulation plot. It can be shown in Figure 1.

The scheduling is to build a mapping relation from simulation event sets to simulation service sets by the scheduling algorithm. Its purpose is to choose the best service composition path under the simulation task limited condition to effectively complete the simulation. In the following part, a simulation task example of artillery firepower attacking is described. In this simulation task plot, artillery firepower attacking step is as follows.

1. The artillery needs to get enemy's position by radar search service.
2. The artillery needs to get enemy's global situation by situation analysis service.
3. The artillery needs to prepare for firing by firing preparation service.
4. The artillery needs to fire allocation scheme by fire allocation service.

**Figure 2:**
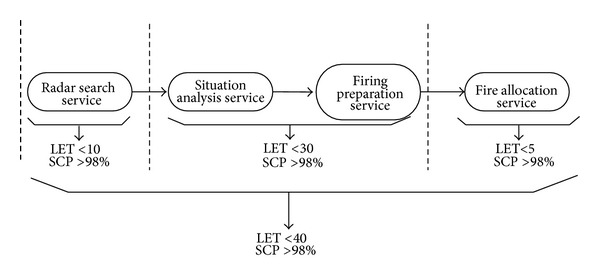
The service composition model under condition.

In order to improve timeliness and the probability of success in simulation task, the probability of success and response time should be constrained. In the first phase, the longest echo time (LET) of radar service should be less than 10 seconds and the success call probability (SCP) should be more than 98%. In the second phase, for situation analysis service and firing preparation service, LET is required less than 30 seconds and SCP is required more than 98%. In the third phase, for situation analysis service and firing preparation service, LET is required less than 30 seconds and SCP is required more than 98%. For the whole of simulation task, LET is less than 40 seconds and SCP is required more than 98%. It can be shown in Figure 2.

### 2.2. The Description of Service Scheduling Model

Simulation task plot is used to arrange role completion action in specific time and position and it is important measure for workflow service scheduling. In fact, service scheduling is to optimize the simulation service composition, and service scheduling model can be simply described as the relation of simulation service composition optimization according to the simulation plot. Therefore, service scheduling optimization model can be described as: *P* = {*S*, *P*, *C*} wherein *S* is representative of the service sets according to the event in plot, *S* = {*S*_1_, *S*_2_, *S*_3_,…, *S*_*n*_}; *P* is representative of the mapping from the event relation to simulation service relation; *C* is representative of the constrained condition in the whole process.

### 2.3. The Optimization of Service Composition Path

**Figure 3:**
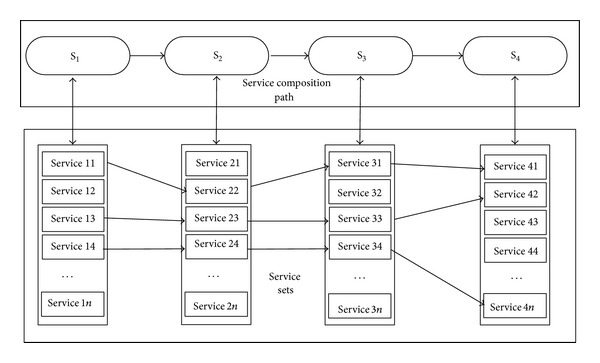
Service composition path choosing.

The service composition path is selected one by one from simulation service sets to form a service composition path to complete simulation task. It can be shown in Figure 3.

From Figure 3, we can see that many paths can be selected by the methods of exhaustion, intelligence algorithm, and so forth to compile statistics quality of service and get the service composition path. However, these methods cannot ensure that the path is the best choice, because these methods are only considering the ideal condition without considering unexpected exception in practice.

## 3. The Strategy of Multipath Parallel Allocation for Service

**Figure 4:**
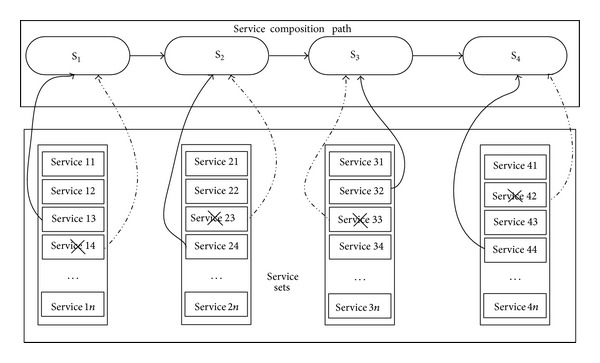
Failed service instead sketch.

When the simulation service is called, various problems may occur in the simulation task workflow during run time and the task may not be completed. In order to solve this problem, many researchers have taken a variety of approaches such as failed service instead, recall service again, and reselection of the path of service composition. The method of failed service instead is to replace the simulation service which is unable to continue to provide the service in process of the simulation task, and then another appropriate simulation service which has the same function in the service sets is called to complete the service function which the exception simulation service should complete. It can be shown in Figure 4.

The method of recall service is to recall service when the simulation service cannot complete the appropriate service function and surpass maximum response time. This method can be applied when the simulation service in service sets is not enough or it requires a higher limitation of simulation to use. Because the time expense for this method is small, if we recall the service again, it will restore the function promptly, so the simulation task workflow may restore the work in the shortest time.

The method of reselection of the path of service composition is to choose a service composition path again under the constrained condition and then run the simulation task again. This method's time expenses are large. Because it means the simulation service in original path should complete the task again in the new service composition path, so this method is used in the situation in which both of the expiration substitution method and recall service method are unable to complete the simulation task.

**Figure 5:**
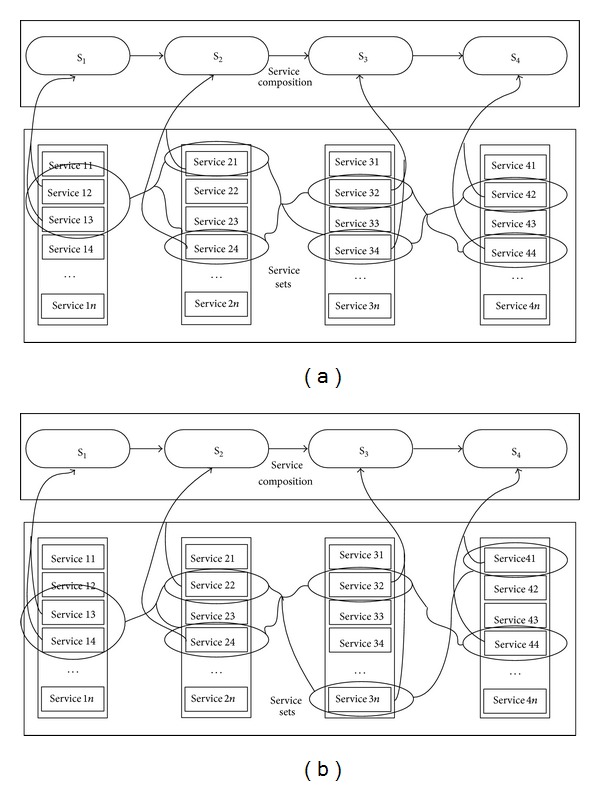
(a) The first path for multiple service parallel allocation. (b) The second path for multiple service parallel allocation.

In fact, although these methods may reduce the influence to some extent on the simulation task workflow when the simulation service makes the mistake, it also takes time expenses as price. This is not permitted for real time simulation task, so it needs to use a more nimble method to solve this problem. In order to improve the probability of success and timeliness in simulation task workflow, this paper proposes an optimization algorithm for multipath service resource parallel allocation to handle service failure to be called in the service composition process, and each path is a parallel distribution services allocation plot. In the practice, various traditional methods such as failed service instead and recall service can be used synthetically. For example, two paths are selected and each node has two parallel services in each path. It can be shown in Figures 5(a) and 5(b).

This method is simultaneously calling many paths, and in each path simulation service simultaneously is called with many parallel services in service sets. The shortest response time of the first path in a number of paths can be selected as the main implementation of the path, in which any service can be selected as a main service when it has the shortest response time. If other main service is failed, other parallel service will be selected as main service. The difference from traditional instead methods is the parallel services running, and the workflow work cannot be effected even if one service is exception. Therefore, it nearly does not spend other expenses. In particular, because many paths run simultaneously, if the service in the main path produces an unexpected result, the service in another path can also run instead of it in time to effectively improve probability of success and timeliness for simulation task.

## 4. The Model of Multipath Service Parallel Allocation

Suppose there exist *n* agents in accordance with *n* service sets in workflow. *S*_*i*_  (*i* = 1,2, 3,…, *n*) represents the service in accordance with the *i*th service agent. *S*_*ij*_  (*j* = 1,2, 3,…, *m*) represents the *i*th service agent in accordance with *m* services in simulation service bus and the *j* represents the *j*th service in *m* services called. Suppose the probability of success service *S*_*ij*_ is *f*(*i*, *j*) and its completion time follows a normal distribution under mean as *u*_*ij*_ and variance as *σ*_*ij*_^2^. When the workflow simultaneously call m service in *S*_*i*_, its probability can be shown in the following formula:

(1) $$\begin{array}{c} \text{Suc}\left(i\right)=1-\overset{m}{\underset{j=1}{\prod }}\left(1-f\left(i,j\right)\right). \end{array}$$

*M*_*i*_ = *Min*⁡(*t*_*i*,1_, *t*_*i*,2_,…, *t*_*i*,*m*_) is used to represent time distribution function in accordance with the *i*th service agent, where *t*_*i*,*j*_ is the *i*th service in *m* services called by service agent. It is normal distribution and can be shown in the following formula:

(2) Ti(t,j)=Suc(Mi≤t)=1−Suc(Mi≥t)=1−Suc[⋂j=1m(ti.j≥t)]=1−∏j=1mSuc(ti,j≥t)=1−∏j=1m[1−Suc(ti,j<t)]=1−∏j=1m[1−Ti,j(t)]=1−∏j=1m[1−12πσi,j∫0te−(t−ui,j)2/2σi,j2dt].

The success probability of simulation service *S*_*i*_ in the *i*th simulation in the waiting time of *d*_*i*_ is as follows:

(3) SucTi(di,i)=Suc(i)∗Ti(t,j)=[1−∏j=1m(1−f(i,j))]×[1−∏j=1m(1−12πσi,j×∫0die−(t−ui,j)2/2σi,j2dt)].

Suppose *n* events according to *n* services in a service composition path. Let *m* = 〈*m*_1_, *m*_2_,…, *m*_*n*_〉, where *m*_*i*_  (*i* = 1,2,…, *n*) represents the number of parallel called services in this node. Suppose simulation service bus could support *A*_*i*_  (*m*_*i*_ ∈ *A*_*i*_) service for the *i*th node, and the system expense in the parallel running *m*_*i*_ services can be shown by the following formula:

(4) $$\begin{array}{c} C_{i}\left(m_{i}\right)=e^{{\left(m_{i}/A_{i}\right)}^{2}}. \end{array}$$

Therefore, the whole process of parallel service running expense can be shown as

(5) $$\begin{array}{c} C\left(m\right)=\overset{n}{\underset{i=1}{\sum }}e^{{\left(m_{i}/A_{i}\right)}^{2}}. \end{array}$$

The ultimate optimization goal is to select proper *m* which is satisfied with constraint condition for service time and probability, and the service running expense is the lowest. Suppose the weighting for the *i*th service in the whole service is *W*_*i*_, ∑_*i*=1_^*n*^ *W*_*i*_ = 1 and the ultimate optimization model can be represented as

(6) Min⁡ (c(m))=Min⁡∑i=1nWie(mi/Ai)2s.t.SucTj(SucT1(d1,m1),SucT2(d2,m2)⋯SucTn(dn,mn))>SCPj0<Tj(d1,d2,…,dn)<LETj.

## 5. Service Scheduling Optimization Based on Quantum Optimization Algorithm 

In fact, the problem of multiple services parallel allocation is service resource scheduling problem which is a NP problem. Many methods have been proposed to solve this problem: GA, PSO, and so forth. However, there always exist many disadvantages in partial convergence or slow optimization. Quantum computation is based on the principal concepts of the quantum theory [13, 14]. Numerous researchers have devoted increasing interests to quantum computation, a novel interdisciplinary field that covers quantum mechanics and information science [15–24]. This paper tries to use a quantum optimization algorithm with multiple chain coding schemes to solve this problem and mainly use four-chain quantum-inspired evolutionary algorithm (FCQIEA) to solve.

### 5.1. Expanded Encoding Method for Quantum Chromosome

**Figure 6:**
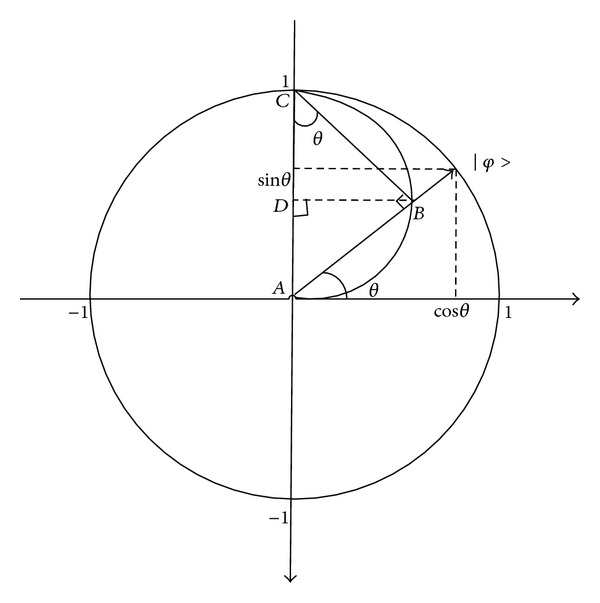
Schematic diagram of the qubit probability amplitude decomposition.

From [24] we can know that multiple chains can be required in Figure 6.

From Figure 6, we can get (7) as follows:

(7) $$\begin{array}{c} |\varphi >={\left[\cos\theta ,\cos\theta \sin\theta ,\sin\theta \sin\theta \right]}^{T}. \end{array}$$

To describe the quantum dynamics behavior objectively, comprehensively, and unambiguously, we can use a new angle *φ* (0 < *φ* < *π*), which is called “supporting role,” to replace *θ* and obtain vector sin*θ* as follows:

(8) $$\begin{array}{c} {\left[\sin\varphi \times \sin\theta ,\cos\varphi \times \sin\theta ,\cos\theta \right]}^{T}. \end{array}$$

Equation (8) also satisfies the normalization condition. In fact, (8) also corresponds to the three-chain encoding method:

(9) $$\begin{array}{c} p_{i}=\left|\begin{array}{c} \sin\varphi_{i1}\sin\theta_{i1}\\ \cos\varphi_{i1}\sin\theta_{i1}\\ \cos\varphi_{i1} \end{array}\right|\cdots \left|\begin{array}{c} \sin\varphi_{\text{in}}\sin\theta_{ij}\\ \cos\varphi_{\text{in}}\sin\theta_{ij}\\ \cos\varphi_{ij} \end{array}\right|. \end{array}$$

Likewise, we can obtain vector sin*φ*sin*θ* by adding the “supporting role” *β* to form the four-chain encoding method as follows:

(10) $$\begin{array}{c} {\left[\cos\beta \times \cos\varphi \times \sin\theta ,\sin\beta \sin\varphi \times \sin\theta ,\cos\varphi \sin\theta ,\cos\theta \right]}^{T},\\ p_{i}=\left|\begin{array}{c} \cos\beta_{i1}\sin\varphi_{i1}\sin\theta_{i1}\\ \sin\beta_{i1}\sin\varphi_{i1}\sin\theta_{i1}\\ \cos\varphi_{i1}\sin\theta_{i1}\\ \cos\theta_{i1} \end{array}\right|\cdots \left|\begin{array}{c} \cos\beta_{ij}\sin\varphi_{ij}\sin\theta_{ij}\\ \sin\beta_{ij}\sin\varphi_{ij}\sin\theta_{ij}\\ \cos\varphi_{ij}\sin\theta_{ij}\\ \cos\theta_{ij} \end{array}\right|. \end{array}$$

We can obtain four optimal solutions, which are expressed as follows:

(11) $$\begin{array}{c} p_{i1}=\left(\cos\beta_{i1}\sin\varphi_{i1}\sin\theta_{i1},\ldots ,\cos\beta_{\text{in}}\sin\varphi_{\text{in}}\sin\theta_{\text{in}}\right)\\ p_{i2}=\left(\sin\beta_{i1}\sin\varphi_{i1}\sin\theta_{i1},\ldots ,\sin\beta_{\text{in}}\sin\varphi_{\text{in}}\sin\theta_{\text{in}}\right)\\ p_{i3}=\left(\cos\varphi_{i1}\sin\theta_{i1},\ldots ,\cos\varphi_{\text{in}}\sin\theta_{\text{in}}\right)\\ p_{i4}=\left(\cos\theta_{i1},\ldots ,\cos\theta_{\text{in}}\right). \end{array}$$

With the same principle, we can get *N* + 1 chains' coding scheme as follows:

(12) pi=|(sinθn)i1(sinθn−1)i1⋯(sinθ2)i1(sinθ1)i1(cos⁡θn)i1(sinθn−1)i1⋯(sinθ2)i1(sinθ1)i1⋮(cos⁡θ3)i1(sinθ2)i1(sinθ1)i1(cos⁡θ2)i1(sinθ1)i1(cos⁡θ1)i1|⋯|(sinθn)ij(sinθn−1)ij⋯(sinθ2)ij(sinθ1)ij(cos⁡θn)ij(sinθn−1)ij⋯(sinθ2)ij(sinθ1)ij⋮(cos⁡θ3)ij(sinθ2)ij(sinθ1)ij(cos⁡θ2)ij(sinθ1)ij(cos⁡θ1)ij|.

### 5.2. Solution Space Transformation

In the quantum evolution progress, all qubits have limited values within  –1 to 1. Thus, we need to transform all qubit values from unit space *I* ^*n*^ = [−1,1]^*n*^ to space *Ω* of the continuous optimization problem (1) by using linear transformation. Each gene value corresponds to an optimization variable in the solution space. If the*j*th qubit on chromosome *p*_*i*_ is [*x*_*ij*_^4^, *x*_*ij*_^3^, *x*_*ij*_^2^, *x*_*ij*_^1^], then the corresponding variables in the solution space are computed as follows:

(13) $$\begin{array}{c} X_{i1}^{j}=0.5\times \left[b_{j}\left(1+x_{ij}\right)+a_{j}\left(1-x_{ij}\right)\right]\\ X_{i2}^{j}=0.5\times \left[b_{j}\left(1+x_{ij}\right)+a_{j}\left(1-x_{ij}\right)\right]\\ X_{i3}^{j}=0.5\times \left[b_{j}\left(1+x_{ij}\right)+a_{j}\left(1-x_{ij}\right)\right]\\ X_{i4}^{j}=0.5\times \left[b_{j}\left(1+x_{ij}\right)+a_{j}\left(1-x_{ij}\right)\right], \end{array}$$

where *i* = 1,2, 3,…, *m* and *j* = 1,2, 3,…, *n*. Thus, each chromosome maps to four approximate solutions of the optimization problem.

### 5.3. Quantum Chromosome Update

Considering that *m* quantum chromosomes are present in the colony and we can obtain 4*m* approximate solutions by solution space transformation, we can then compute the fitness of these approximate solutions and define the solution with the maximum fitness as the current optimum solution in the quantum evolution progress. The chromosome corresponds to the current optimum solution called the optimum chromosome. By computing the fitness, we can obtain both optimum solution and optimum chromosome and subsequently update the colony by using the quantum rotation gate to obtain the optimal solution. In this updated process, the new optimum chromosome can be produced such that the colony can likely evolve. The present study proposes the quantum rotation gate *U* to update the individual qubit as follows:

(14) U=[u11u12u13u14u21u22u23u24u31u32u33u34u41u42u43u44],u11=[cos⁡Δβcos⁡Δφ(cos⁡Δθ−sinΔθcos⁡θsinθ)]T,u12=[sinΔβcos⁡Δφ(sinΔθcos⁡θsinθ−cos⁡Δθ)]T,u13=[sinΔφcos⁡Δθ(sinβsinΔβ−cos⁡βcos⁡Δβ)]T,u14=[cos⁡φsinΔφ(cos⁡βcos⁡Δβ−sinβsinΔβ)]T,u21=[−sinΔβsinΔφ(cos⁡Δθ+sinΔθcos⁡θsinθ)]T,u22=[−cos⁡ΔβsinΔφ(cos⁡Δθ+sinΔθcos⁡θsinθ)]T,u23=[cos⁡Δφcos⁡Δφ(sinβcos⁡Δβ+cos⁡βsinΔβ)]T,u24=[cos⁡φcos⁡Δφcos⁡Δφ(sinβcos⁡Δβ+cos⁡βsinΔβ)]T,u31=−sinΔφcos⁡β,  u32=0,  u33=cos⁡Δφcos⁡Δθ,u34=[sinΔθ(cos⁡φcos⁡Δφ−sinφsinΔφ)]T,u41=u42=0,  u43=sinΔθcos⁡φ,  u44=cos⁡Δθ,U[cos⁡βsinφsinθsinβsinφsinθcos⁡φsinθcos⁡θ] =[cos⁡⁡(β+Δβ)sin⁡(φ+Δφ)sin⁡(θ+Δθ)sin⁡(β+Δβ)sin⁡(φ+Δφ)sin⁡(θ+Δθ)cos⁡⁡(φ+Δφ)sin⁡(θ+Δθ)cos⁡⁡(θ+Δθ)].

### 5.4. Mutation Operation

Quantum nongate is applied to exchange the probability amplitudes to avoid local optimal solution in a certain qubit as follows:

(15) $$\begin{array}{c} \left[\begin{array}{cc} 0 & 1\\ 1 & 0 \end{array}\right]\left[\begin{array}{c} \cos\theta \\ \sin\theta \end{array}\right]=\left[\begin{array}{c} \sin\theta \\ \cos\theta \end{array}\right]. \end{array}$$

Such influence as expressed in (15) can be considered as the phase mutation of a qubit, in which *θ* is mutated to (*π*/2) − *θ*. In this case, a quantum nongate *V* is proposed to mutate the quantum as follows:

(16) $$\begin{array}{c} V=\left[\begin{array}{cccc} \tan\beta \;\text{cot}\varphi \;\text{cot}\theta & 0 & 0 & 0\\ 0 & \tan\beta \;\text{cot}\varphi \;\text{cot}\theta & 0 & 0\\ 0 & 0 & \tan\varphi \;\text{cot}\theta & 0\\ 0 & 0 & 0 & \tan\theta \end{array}\right]. \end{array}$$

### 5.5. The Procedure of FCQIEA

It can be summarized as follows.

**Step 1 (initialize the population)**

Let the current generation *t* = 0; generate an initial population *Q*(*t*) = {*q*_1_^*t*^, *q*_2_^*t*^,…, *q*_*m*_^*t*^}, which has *m* individual qubits. Set the magnitude of the rotational angle |Δ*β*| = *β*_0_, |Δ*φ*| = *φ*_0_, and |Δ*θ*| = *θ*_0_, respectively. Set *p*_*m*_ as the mutation and Max_gen as the maximum generation.

**Step 2 (transform the solution space)**

Four approximate solutions in each chromosome are transformed from the unit space *I* ^*n*^ = [−1,1]^*n*^ to the solution space *Ω* of the continuous optimization problem (1); thus, the set of approximate solution *X*(*t*) can be obtained.

**Step 3 (compute the fitness)**

By computing the fitness of 4*m* approximate solutions, obtain the best solution *BestX* in the current solution and the best chromosome *BestC* in the current chromosome. Store *BestX* as the global optimum solution *GX* and store *BestC* as the global optimum chromosome *GC*.

**Step 4 (set *t* = *t* + 1)**

Update and mutate *Q*(*t* − 1). Calculate the new population *Q*(*t*).

**Step 5**

Transform the solution space again and obtain a set of approximate solution *X*(*t*).

**Step 6**

By computing the fitness of *Q*(*t*), determine the current optimum solution *BestX* and the current optimum chromosome *BestC*. If fit(*BestX*) < fit(*GX*), then update the current optimum solution *BestX* = *GX*; at the same time, update the current optimum chromosome *BestC* = *GC* to avoid population degradation. Otherwise, let *GX* = *BestX* and *GC* = *BestC* so that the algorithm approaches the global optimum solution.

**Step 7**

If the algorithm does not converge and if *t* < Max_gen, then go back to Step 4 until the algorithm becomes convergent or until *t* > Max_gen.

## 6. Simulation Experiment and Analysis 

### 6.1. Comparisons of the Different Optimization Result

Under the hypothesis condition as Figure 2, four events are in accordance with service as *S*_1_, *S*_2_, *S*_3_, and *S*_4_, and the specific constraint conditions can be shown as in Table 1.

**Figure 7:**
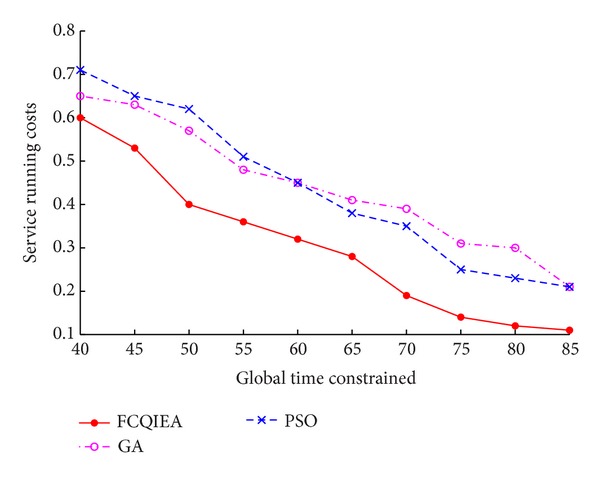
Comparison of the optimization result with different algorithms.

In order to compare the different optimization results between different optimization algorithms, FCQIEA, GA, and PSO, optimize the service scheduling, respectively. In both of FCQIEA and GA, the generation set is 100 and intersection probability and mutation set are 0.6 and 0.1, respectively. The number of coding chains is 4, and maximum rotate angle set Δ*θ* = 0.04*π*. In PSO, regulatory factor set *w* = 0.5 and study factor set *C*_1_ = 0.7 and *C*_2_ = 1.2. In order to avoid the influence of random factors, we performed the calculation 10 times in each algorithm and obtained the average value as the optimization result. The global constraint time of each service node is added with 5 seconds as base unit; the optimization result can be shown in Figure 7.

From Figure 7, obviously, FCQIEA has best performance compared with GA and PSO and its service running costs most rapid decline than other algorithms.

### 6.2. Comparisons of the Service Allocation Strategy

**Figure 8:**
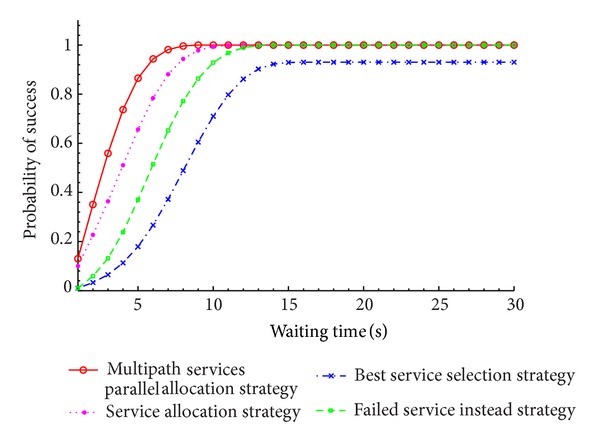
The probability of success of different service allocation strategy.

The different service allocation strategy, that is, selection best service, failed service instead, services parallel allocation, and multipath services parallel allocation, is compared under the constraint condition in Table 1. The failed service is produced randomly with probability of 1%. The failed service instead strategy is that the service can be called failure when waiting time is beyond the 3 times of *δ* (3 times *δ* rule) service execution time and it should select another service to replace the failed service. In service parallel allocation strategy, 20 percent of service sets are randomly selected as parallel execution service. In the multipath service parallel allocation strategy, the number of path set 3 and 20 percent of service sets are randomly selected as parallel execution service. After long running time, the relation between the probability of success and execution of different service allocation strategy is investigated. It can be shown in Figure 8.

**Figure 9:**
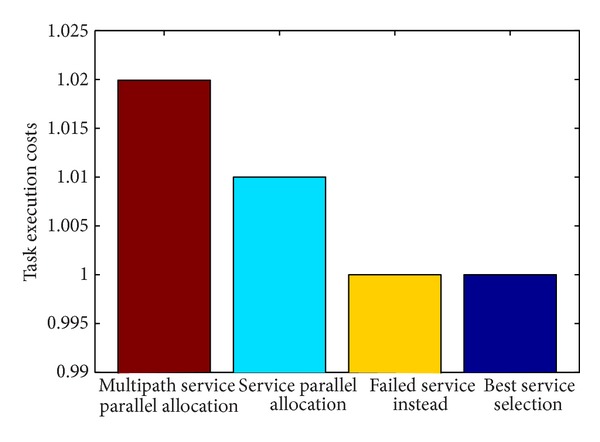
Comparison of task execution costs with different service allocation strategy.

Different service execution costs can be shown in Figure 9.

From analysis, it can be found that the probability of success of multipath service parallel allocation strategy is obviously higher than other strategies; however, it also results in extra system costs than other service allocation strategies.

### 6.3. The Effect of Probability of Success from the Number of Paths

**Figure 10:**
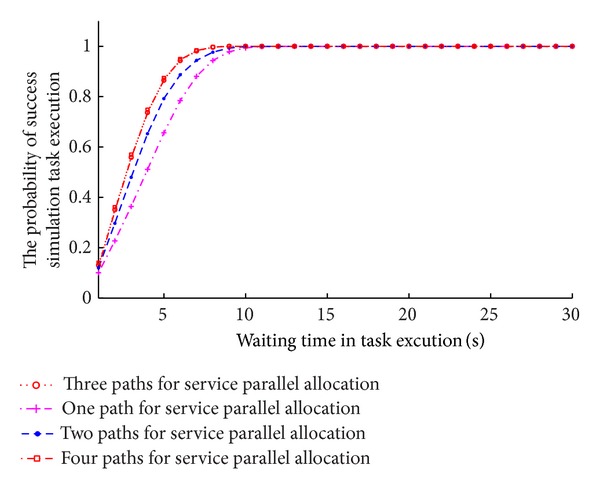
Comparison of the effect for the probability of success simulation task with different paths.

**Figure 11:**
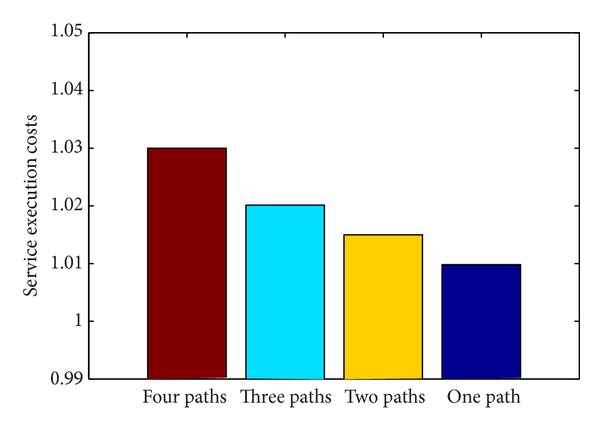
Comparison of simulation task execution costs among different paths.

Service parallel allocation and multipath services parallel allocation are used to execute simulation task and the number of paths is 1, 2, 3, and 4. The failed service is produced randomly with probability of 1%. After running in long time, the relation between the probability of success and execution time is investigated. It can be shown in Figures 10 and 11.

From Figures 10 and 11, we can get the conclusion that we can improve probability of success by adding the number of service paths in service parallel allocation, but it will not always get the best probability of success when increasing the number of service paths, and there exist the best value. From the simulation experiment, it can be seen that the probability of success for completion task is improved obviously with the numbers of 2 and 3, but when the number of path set is 4, the probability of success for completion task is not improved and the simulation execution costs are raised. Therefore, three paths are proper.

## 7. Conclusions

In military modeling and simulation field, it is important to improve the probability of success and timeliness in simulation task workflow. This paper established the multipath service parallel allocation optimization mathematical model to investigate service scheduling optimization algorithm in which FCQIEA is used. Through the simulation experiment, this paper investigates the effect for the probability of success in simulation task workflow from different optimization algorithm, service allocation strategy, and path number, and the simulation result shows that the optimization algorithm for multipath service resource parallel allocation is an effective method to improve the probability of success and timeliness in simulation task workflow. In the next step, multipath service parallel allocation will be used in military modeling and simulation field, and this theory can be improved in practice.

## Figures and Tables

**Table 1:** Service constrained condition.

Constrained condition	Service node			
	*S*_1_	*S*_2_	*S*_3_	*S*_4_
Execution time distribution	[5 s, 15 s] uniform distribution	[10 s, 20 s] uniform distribution	[10 s, 25 s] uniform distribution	[2 s, 8 s] uniform distribution

Probability of success	A random number from 0.94 to 1	A random number from 0.94 to 1	A random number from 0.92 to 1	A random number from 0.93 to 1

The number of parallel services	30	20	30	20

The maximum waiting time	10	30		5
	40			

The minimum probability of success	98%	98%		98%
	98%			

*W*_*i*_	0.2	0.3	0.1	0.4
